# Successful distal splenic artery embolization in a patient with chronic lymphocytic leukemia complicated by thrombocytopenia and splenic artery aneurysm

**DOI:** 10.1016/j.radcr.2026.07.037

**Published:** 2026-08-28

**Authors:** Marisa Patel, Julia Hanna, Reem Yassine, Christopher Zarour

**Affiliations:** a Michigan State University College of Osteopathic Medicine, 965 Wilson Rd, East Lansing, MI, 48824, USA; b Lake Erie College of Osteopathic Medicine, 1858 W Grandview Blvd, Erie, PA, 16509, USA; c Trinity Health Oakland Hospital, Wayne State University Diagnostic Radiology, 44405 Woodward Ave, Pontiac, MI, 48341, USA

**Keywords:** Splenic artery aneurysm (SAA), Splenic artery embolization (SAE), Thrombocytopenia, Distal splenic artery embolization, Chronic lymphocytic leukemia (CLL), Splenomegaly

## Abstract

Splenic artery aneurysms, the most common visceral artery aneurysms, are diagnosed when the diameter of the splenic artery reaches greater than 50% of its normal diameter. Nonruptured, true splenic artery aneurysms are typically treated endovascularly if greater than or equal to 3 cm. There is a risk of rupture of the aneurysm when it is greater than 2-3 cm. Our case consists of a 66 year-old male with a past medical history of chronic lymphocytic leukemia complicated by thrombocytopenia and neutropenia who presented for his annual follow up and restaging of his chronic lymphocytic leukemia with no new symptoms. Initial computed tomography of the chest, abdomen, and pelvis with contrast demonstrated severe splenomegaly, which had increased from prior exam, and a mild aneurysmal dilation of the thoracic aorta with a maximum transverse diameter of 4.2 cm. Subsequent CT angiography of the chest with and without contrast 2 months later revealed a partially imaged 4.3 cm splenic artery aneurysm. A follow-up contrast-enhanced CT of the abdomen and pelvis demonstrated an interval increase in size of the distal splenic artery aneurysm, now measuring 4.6 × 5.5 cm. A consult was then made to Vascular and Interventional Radiology for endovascular embolization. The patient underwent successful splenic artery embolization and was transfused 2 units of platelets before discharge. This article highlights a rare case of a large 5.5 cm distal splenic artery aneurysm successfully treated with coil embolization using the front door-back door embolization technique in the clinical setting of background chronic lymphocytic leukemia, treating both the splenic artery aneurysm and hypersplenism-induced thrombocytopenia. Despite the bleeding risk with embolization and thrombocytopenia, the patient was successfully treated with minimal bleeding and no major complications like rupture or vascular injury, with subsequent improvement of thrombocytopenia by decreasing splenic sequestration of platelets.

## Introduction

Splenic artery aneurysms (SAA) are the most common visceral artery aneurysms and are diagnosed when the diameter of the splenic artery reaches greater than 50% of its normal diameter, which is typically 0.43-0.49 cm [1]. SAAs typically occur distally as saccular aneurysms, which are best treated with endovascular coiling [1]. Nonruptured, true splenic artery aneurysms are typically treated endovascularly if greater than or equal to 3 cm [2]. The risk of rupture increases when the aneurysm is larger than 2-3 cm, indicating need for intervention. Risk factors for true aneurysm formation include conditions that weaken the integrity of arterial vessel walls, such as age, hypertension, atherosclerosis, connective tissue disorders, and trauma [1]. Other risk factors include portal hypertension, increased blood flow and volume to the spleen, splenomeglay, liver transplantation, female sex, and pregnancy [1]. Our patient’s risk factors included age, hypertension, splenomegaly and history of malignancy. In the setting of splenomegaly due to CLL, a subsequent increase in blood flow and volume to the spleen likely contributed to arterial wall weakening and eventual aneurysm formation. Furthermore, the case was complicated by thrombocytopenia secondary to hypersplenism. Partial splenic artery embolization is a less invasive technique compared to splenectomy for the treatment of thrombocytopenia from splenic sequestration, especially in cancer patients requiring systemic chemotherapy that can further exacerbate the thrombocytopenia [3]. A common technique used to treat splenic artery aneurysms is the front door and back door technique, where the “back door” or efferent artery is embolized, followed by the “front door” or afferent artery [4]. This technique assures the artery is adequately embolized in the case of collateral flow that could continue to supply the vessel [4]. This case highlights a successful endovascular intervention of a large 4.6 × 5.5 cm SAA using the front door and back door technique in the setting of thrombocytopenia and splenomegaly secondary to chronic lymphocytic leukemia (CLL), with subsequent improvement of the thrombocytopenia following intervention.

## Case report

### Patient information

A 66-year-old male with B-cell CLL, not in remission and complicated by thrombocytopenia and neutropenia, presented for his annual follow-up and CLL restaging. His medical history was also significant for chronic kidney disease, hypothyroidism, essential hypertension, chronic deep vein thrombosis treated with therapeutic enoxaparin, benign prostatic hyperplasia, and carcinoma in situ of the facial skin. Family history was significant for maternal breast cancer, paternal lung cancer, and hypertension in both parents. He denied tobacco, alcohol, and illicit drug use. Past surgical history includes appendectomy, skin biopsies, and Mohs surgery. He was asymptomatic at the time of his annual visit. Initial laboratory evaluation demonstrated thrombocytopenia with a platelet count of 75 k/mcL (reference range 140-450 k/mcL). During routine restaging, computed tomography angiography (CTA) of the chest incidentally identified a distal splenic artery aneurysm, prompting CTA of the abdomen and pelvis for further characterization. CTA of the abdomen and pelvis demonstrated a large distal splenic artery aneurysm measuring 4.6 × 5.5 cm. The case was reviewed by Interventional Radiology, and the decision was made to proceed with endovascular embolization. Anticoagulation was held 48 hours prior to the procedure, and the patient underwent successful splenic artery embolization.

### Computer tomography findings

**Fig. 1:**
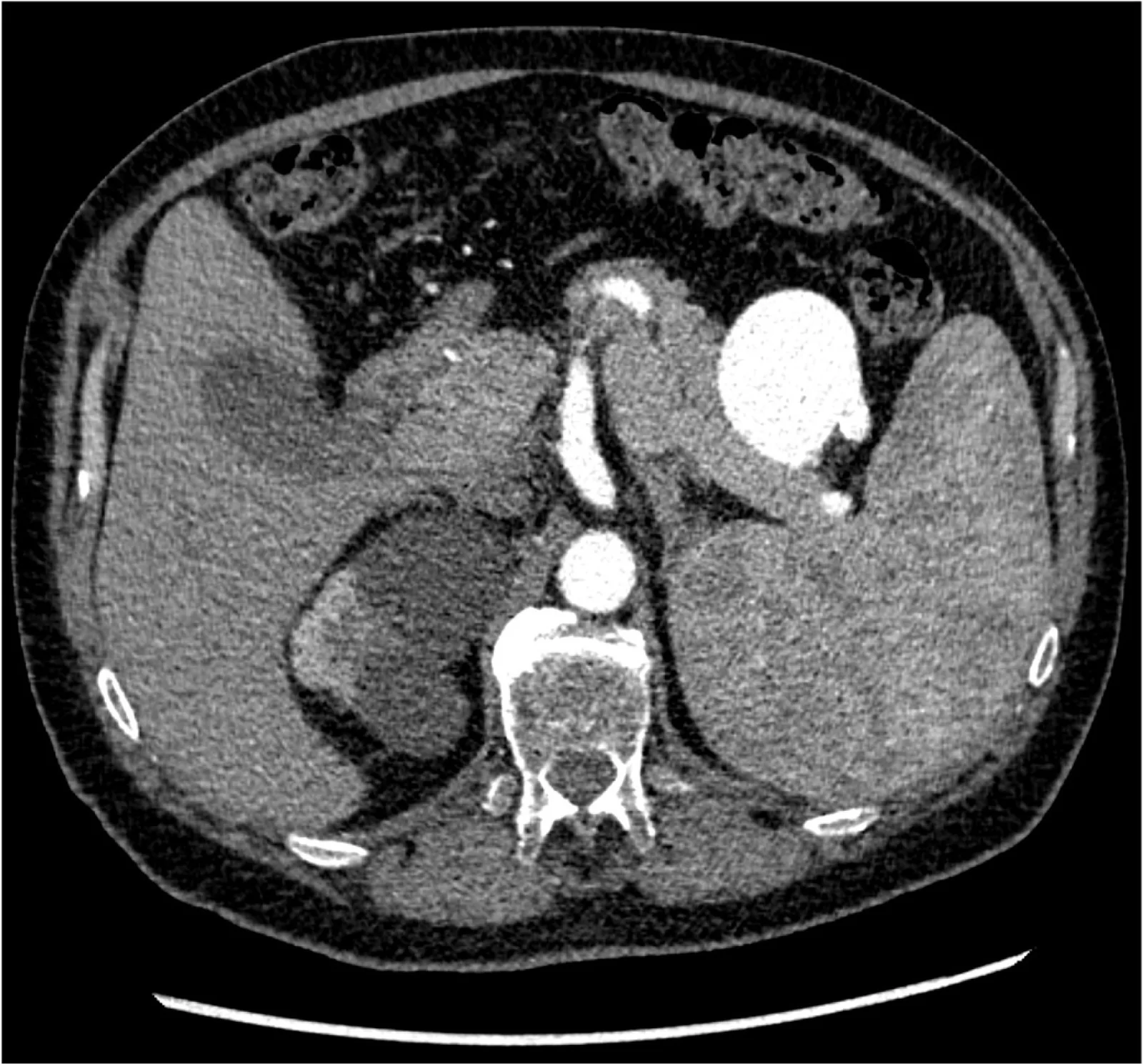
CT angiogram chest shows splenomegaly and partially imaged 4.3 cm splenic artery aneurysm within the splenic hilum.

**Fig. 2:**
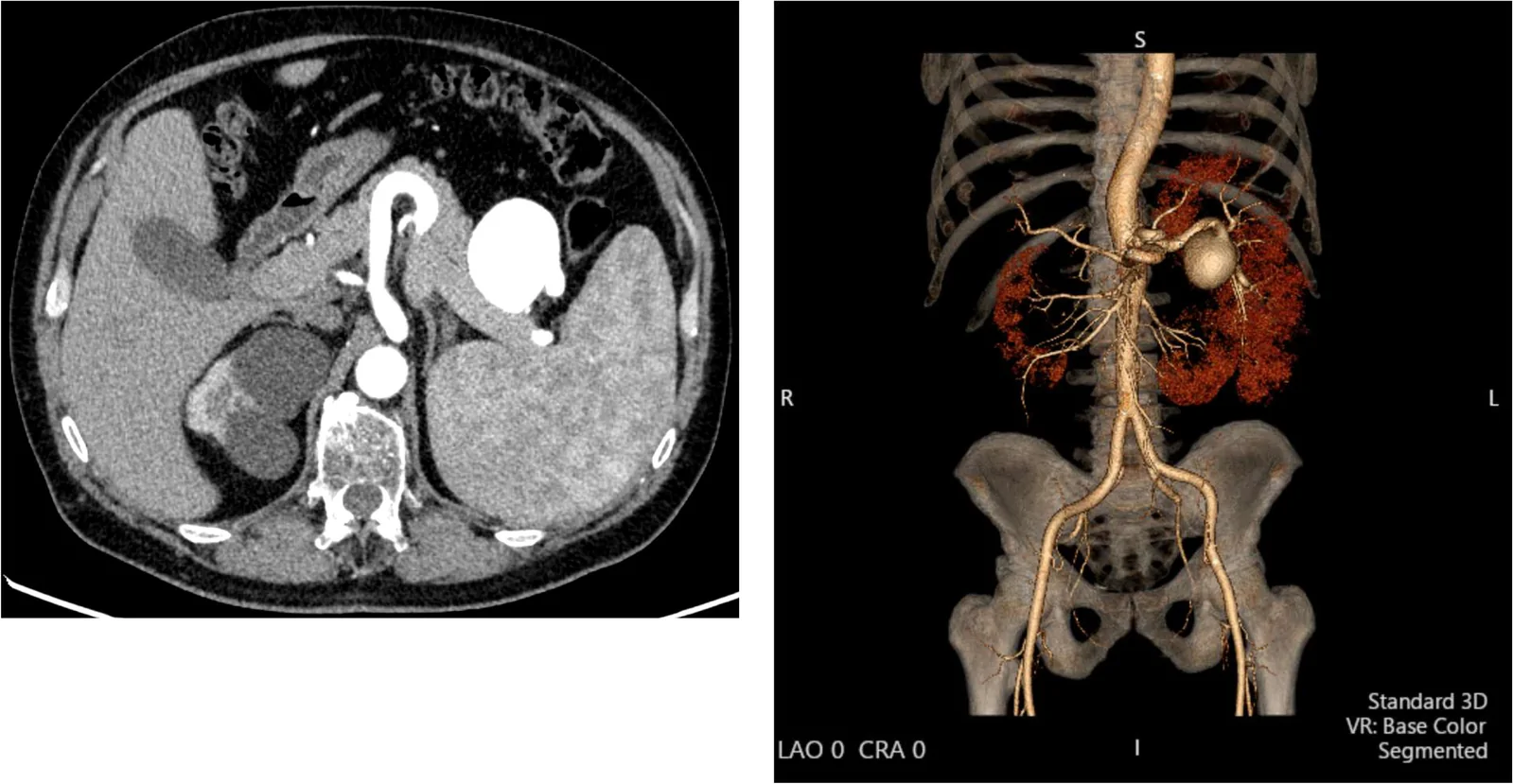
Interval increase in size of the distal, splenic artery aneurysm, now measuring 4.6 × 5.5 cm. Given this interval increase in size, vascular and interventional radiology was consulted for embolization. Stable splenomegaly.

Computed tomography (CT) of the chest, abdomen, and pelvis with contrast demonstrated severe splenomegaly increased from prior exam and mild aneurysmal dilation of the thoracic aorta with a maximum transverse diameter of 4.2 cm. Follow up CT angiography of the chest with and without contrast 2 months later revealed a partially imaged 4.3 cm splenic artery aneurysm (Fig. 1). Further, a CECT of the abdomen and pelvis 2 months later demonstrated an interval increase in size of the distal splenic artery aneurysm now measuring 4.6 × 5.5 cm (Fig. 2). Vascular and Interventional Radiology (VIR) was consulted for embolization.

### Intervention and outcome

The case was reviewed by VIR and the decision was made to proceed with endovascular embolization with goals of a dual therapeutic strategy, treating both the SAA and thrombocytopenia. Anticoagulation was held for 48 hours prior to the procedure. The patient underwent successful splenic artery embolization using the front door and back door (aka sandwich) technique, in which both the outflow and inflow vessels were embolized to prevent possible collateral flow from supplying the affected vessel. This allows treatment of both the aneurysm and thrombocytopenia through partial splenic embolization, diminishing splenic sequestration of platelets.

**Fig. 3:**
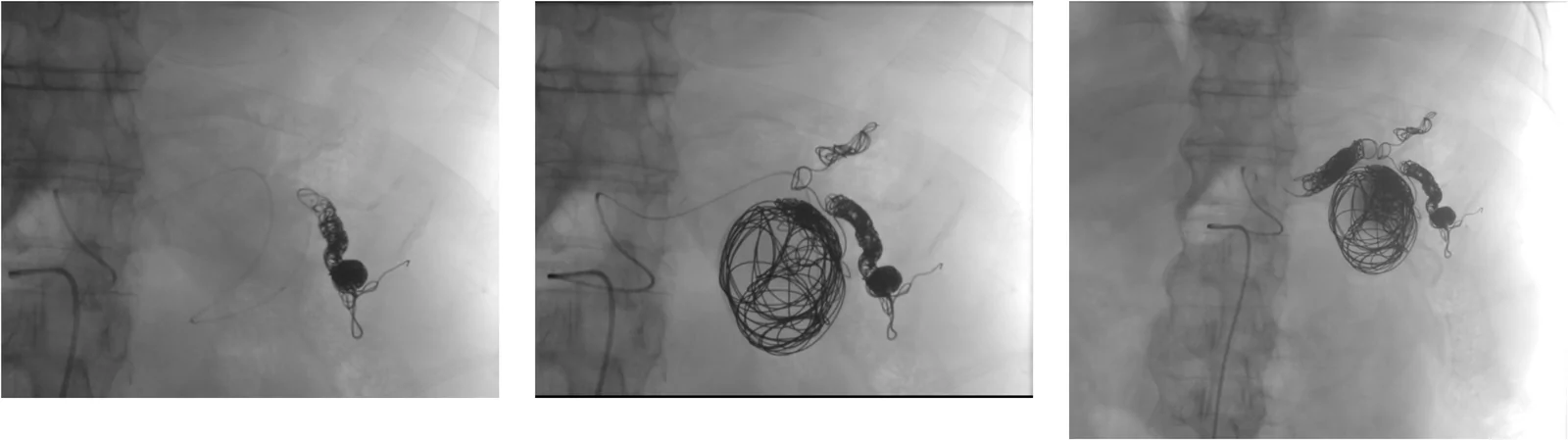
Step 1: outflow vessel catheterization with multiple 8 mm deployable coils. Step 2: the microcatheter was retracted into the aneurysm sac which was then packed with multiple 40 mm coils and approximately 5 cc of a Gelfoam slurry. Step 3: inflow vessel catheterization with multiple 8 mm deployable coils.

Using a reverse curve 5 French catheter, the celiac artery was catheterized and angiogram demonstrated conventional branching anatomy with a large distal splenic artery aneurysm as seen on comparison CTA. A 0.035 wire was advanced through the catheter into the medial aspect of the splenic artery, and the catheter was removed over the wire. The short 5 French sheath and a 5 French by 45 cm sheath was advanced over the wire and placed into the proximal splenic artery. Through this sheath, a 4 French glide catheter was advanced into the middle aspect of the splenic artery. Through the base catheter a 2.8 microcatheter was advanced into the aneurysm, and the outflow vessel was catheterized. Embolization of the outflow vessel was performed with multiple 8 mm deployable coils. Following embolization of the outflow vessel, the microcatheter was retracted into the aneurysm sac, and the aneurysm sac was packed with multiple 40 mm coils and approximately 5 cc of Gelfoam slurry. The microcatheter was retracted into the inflow vessel and multiple 8 mm coils were deployed. Post embolization angiogram demonstrated satisfactory hemostasis of the aneurysm (Fig. 3).

**Fig. 4:**
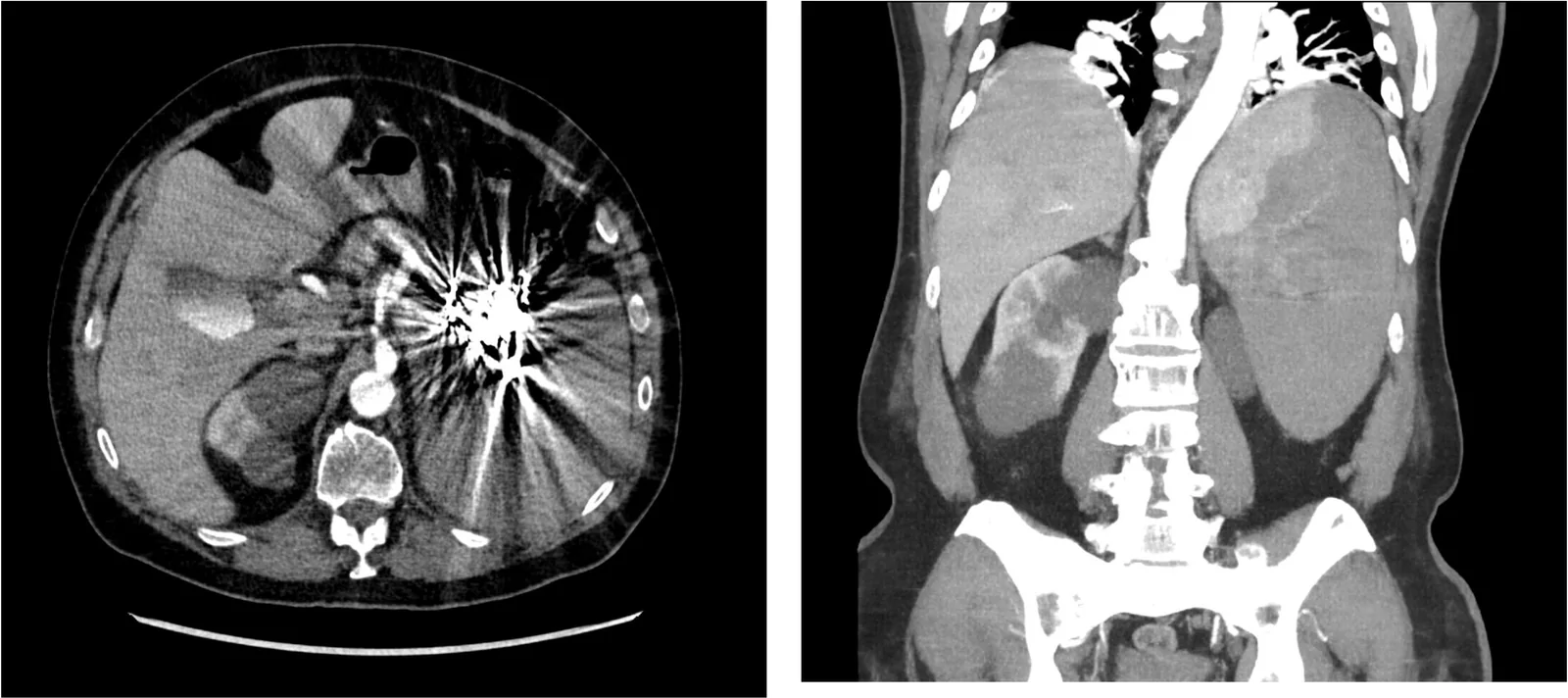
Follow-up CT angiogram axial and coronal views 1 day post procedure demonstrate expected evolving post embolization changes to the splenic parenchyma including a large hypodense nonenhancing region within the majority of the spleen.

**Fig. 5:**
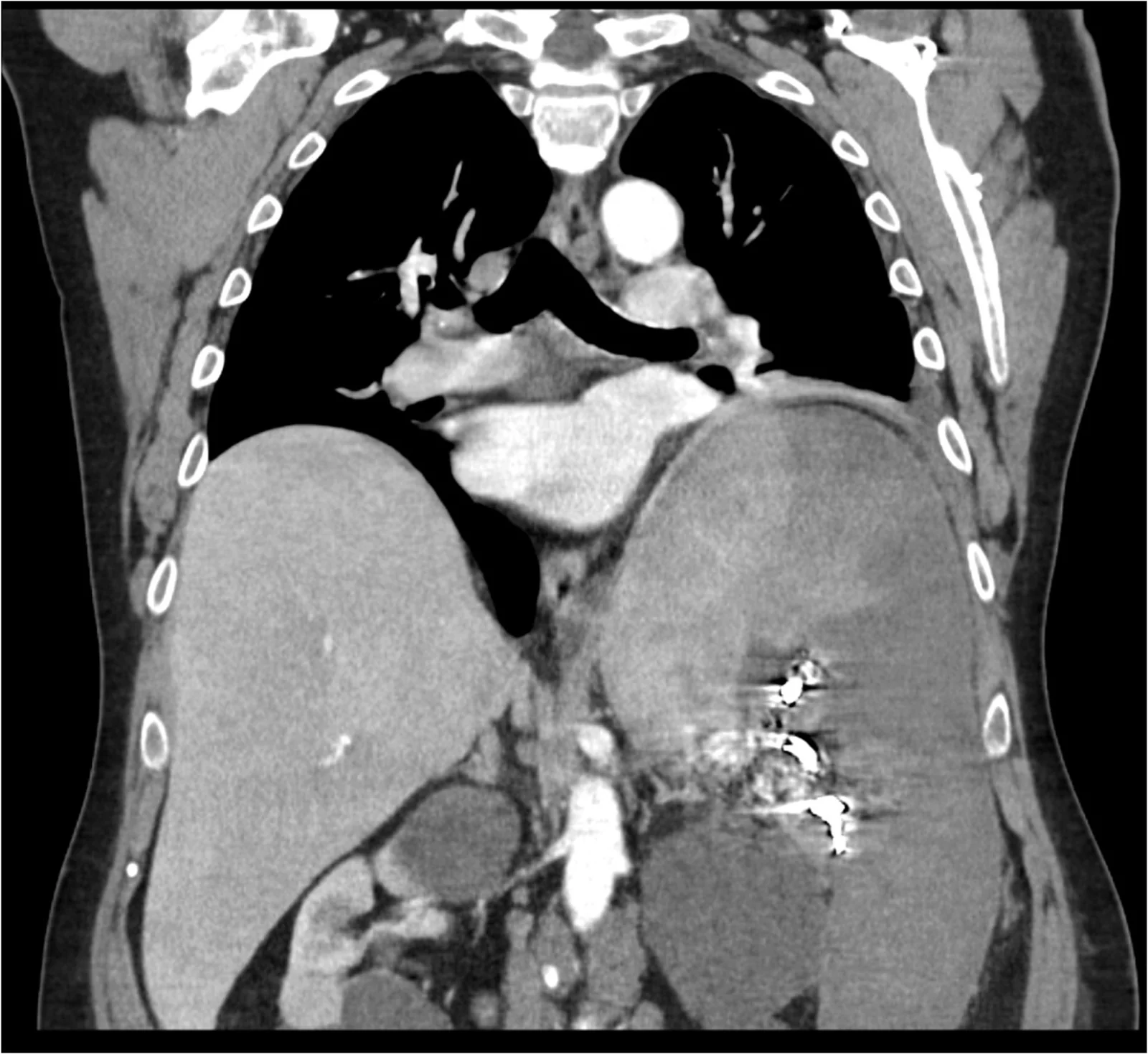
Follow up CT chest with contrast coronal view 21 days post embolization procedure shows continued expected evolving post embolization changes to the spleen including volume reduction and hypodense nonenhancement.

The patient’s thrombocytopenia resolved within one month following the procedure, normalizing to 220 (reference 140-450 k/mcL). Follow up CT angiogram 1 day after the procedure demonstrated a large hypodense nonenhancing region within the majority of the spleen (Fig. 4), indicating the area of partial splenic embolization. Additional follow up CT chest 21 days post procedure redemonstrated splenic hypodense nonhenancement along with splenic volume reduction, indicating successful partial splenic artery embolization and subsequent reduction in splenic volume (Fig. 5).

## Discussion

SAAs are typically treated endovascularly when measuring ≥ 3 cm, with increased risk of rupture if larger than 2-3 cm [1,2]. SAAs typically occur distally as saccular aneurysms, which are best treated with endovascular coiling [1]. However, our patient’s case was complicated by thrombocytopenia secondary to CLL, which increased the risk of bleeding during endovascular intervention, in addition to the elevated risk of aneurysmal rupture associated with the aneurysm’s large size (5.5 cm). Given the increased risk of aneurysmal rupture, selecting the most appropriate intervention is critical.

When selecting an endovascular technique, preservation of the parent artery and its branches is essential to minimize the risk of end-organ ischemia and visceral injury [5]. Aneurysms involving the main splenic artery are preferably treated with coil embolization using 0.035-inch to 0.018-inch coils [5].

This case is unique because it describes the management of a large (5.5 cm) distal splenic artery aneurysm in a patient with thrombocytopenia secondary to CLL. The combination of a high rupture risk due to aneurysm size and an elevated procedural bleeding risk posed a significant therapeutic challenge, requiring careful multidisciplinary planning to balance procedural safety with timely intervention. Close collaboration between the Hematology and Interventional Radiology teams was essential for procedural planning and peri-procedural management. In this patient, endovascular embolization was selected because it offered a less invasive alternative to open surgical repair while providing effective aneurysm exclusion. The patient underwent successful splenic artery embolization with peri-procedural transfusion of two units of platelets and was subsequently discharged home in stable condition. The embolization decreased the risk of SAA rupture and may have offered an additional therapeutic benefit in the management of the patient’s thrombocytopenia. However, improvement in hematologic parameters in patients with CLL is multifactorial and cannot be attributed solely to embolization.

Although SAA embolization is primarily performed to reduce the risk of aneurysmal rupture, previous reports have suggested that partial splenic embolization may provide additional benefit in selected patients with hematologic disorders complicated by hypersplenism and thrombocytopenia, including CLL. Previous studies have reported associations between partial splenic embolization and subsequent improvements in peripheral blood counts, as well as a 60%-70% reduction in splenic size during follow-up [6]. These findings suggest that splenic embolization may have a therapeutic role in carefully selected patients with CLL-associated hypersplenism and thrombocytopenia, although larger studies are needed to better define its efficacy. In one published case, follow-up over a two-year period demonstrated progressive improvement in peripheral blood counts after embolization. Specifically, the red blood cell count increased from 1.9 to 4.8 cell/mcl and the platelet count from 67 to 333 platelet/mcl. However, this temporal association does not establish a direct causal relationship [6]. Evidence regarding SAA embolization in patients with thrombocytopenia remains limited. This case contributes to the growing body of literature suggesting that endovascular treatment can be performed safely in carefully selected patients with hematologic disorders, particularly when multidisciplinary planning is employed. Additional studies are needed to better characterize both procedural outcomes and hematologic effects.

Although this case demonstrates the successful use of endovascular embolization in a patient at elevated procedural risk, long-term imaging surveillance and hematologic follow-up remain essential to assess aneurysm exclusion, monitor for delayed complications, and further characterize long-term outcomes.

## Conclusion

Splenic artery aneurysms (SAA) are the most common visceral artery aneurysms. SAA carries an increased risk of rupture, especially when the aneurysm is larger than 2-3 cm, which indicates a need for intervention. Although the patient in this case has been diagnosed with a hematological condition, such as CLL, essential hypertension, is advanced in age, and underwent previous chemotherapy treatment, this case highlights that endovascular embolization can be safely performed and can serve a dual therapeutic role by preventing SAA ruptures and improving thrombocytopenia. This report also calls attention to the importance of approaching high-risk patients with exponential care and careful consideration. Continued monitoring and imaging will be essential in ensuring the patient has a positive long-term outcome without major complications. Although there is limited literature in regard to performing splenic artery embolization, particularly in individuals with increased risk for thrombocytopenia and those diagnosed with hematological pathologies, this report acts as a bridge for an expansion of literature in regard to this topic, and as literature to advocate for the efficacy of this procedure and its additional benefit of improvement in thrombocytopenia.

## Patient consent

Written informed consent was obtained from the patient for publication of this case report.
